# Missed Opportunities? Covid-19, Biosecurity and One Health in the United Kingdom

**DOI:** 10.3389/fvets.2020.00577

**Published:** 2020-08-28

**Authors:** Gareth Enticott, Damian Maye

**Affiliations:** ^1^School of Geography and Planning, Cardiff University, Cardiff, United Kingdom; ^2^Countryside and Community Research Institute, University of Gloucestershire, Cheltenham, United Kingdom

**Keywords:** COVID-19, biosecurity, One health, animal health, social science

## Abstract

Whatever we read about Covid-19, the word unprecedented is not far away: whether in describing policy choices, the daily death tolls, the scale of upheaval, or the challenges that await a readjusting world. This paper takes an alternative view: if not unpredictable, the crisis unfolding in the United Kingdom (UK) is not unprecedented. Rather, it is foretold in accounts of successive animal health crises. Social studies of biosecurity and animal disease management provide an “anticipatory logic” - a mirror to the unfolding human catastrophe of Covid-19, providing few surprises. And yet, these accounts appear to be routinely ignored in the narrative of Covid-19. Do social studies of animal disease really have no value when it comes to guiding and assessing responses to Covid-19? To answer this question, we describe the narrative arc of the UK's approach to managing Covid-19. We then overlay findings from social studies of animal disease to reveal the warnings they provided for a pandemic like Covid-19. We conclude by reflecting on the reasons why these studies have been paid minimal attention and the extent to which the failure to learn from these lessons of animal health management signals a failure of the One Health agenda.

## Introduction: an Unprecedented Crisis?

Unprecedented. Whatever we read about Covid-19, the word unprecedented is not far away: whether in describing policy choices, the daily death tolls, the scale of upheaval, or the challenges that await a readjusting world. This paper takes an alternative view: if not unpredictable, the crisis unfolding in the United Kingdom (UK) is not unprecedented. Rather, it is foretold in accounts of successive animal health crises. In the UK at least, social studies of biosecurity and animal disease management provide an “anticipatory logic” - a mirror to the unfolding human catastrophe of Covid-19, providing few surprises. And yet, these accounts appear to be routinely ignored in the narrative of Covid-19 or as social scientists have sought to claim a place at the disease control table alongside traditional forms of expertise like epidemiology. Do social studies of animal disease really have no value when it comes to guiding and assessing responses to Covid-19? Following Rosenberg's [(1), p. 3] description of epidemics as a “dramaturgic event,” we answer this question by firstly describing the narrative arc of the UK's approach to managing Covid-19. We then overlay findings from social studies of animal disease to reveal the warnings they provided for a pandemic like Covid-19. We then reflect on the reasons why these studies have been paid minimal attention and the extent to which the failure to learn from these lessons of animal health management signals a failure of the One Health agenda.

## COVID-19 in the UK

**Figure 1:**
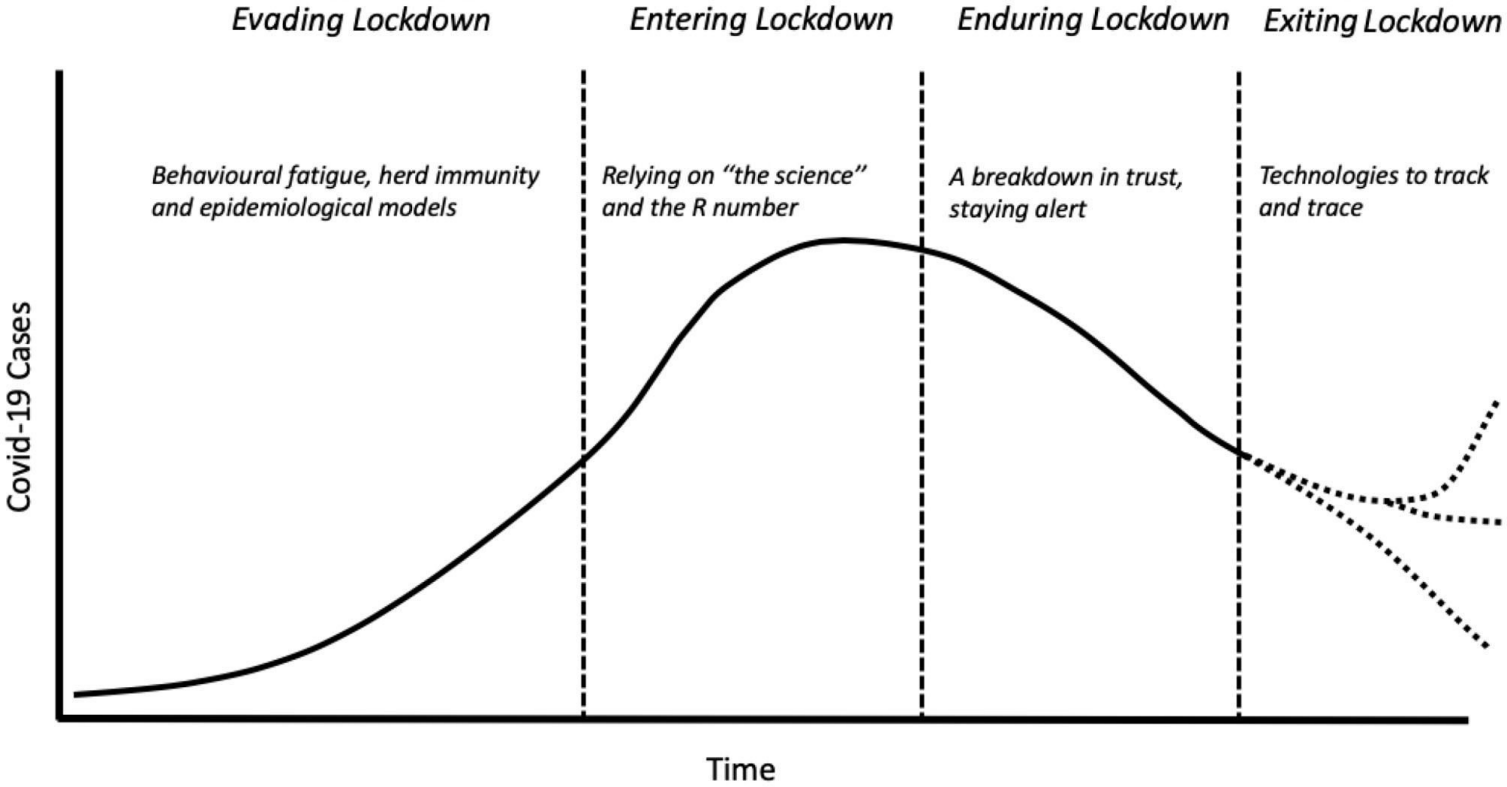
Key stages of the Covid-19 epidemic curve in the UK. Dotted lines reflect the uncertain paths the Covid-19 epidemic may take in future.

Rosenburg [(1), p. 2] describes epidemics as a dramaturgic form, following a plot line “of increasing revelatory tension, move to a crisis of individual and collective character, then drift toward closure.” In doing so, this narrative arc “illuminat[es] fundamental patterns of social value and institutional practice” (ibid.). The responses to Covid-19 in the UK share Rosenberg's archetypal epidemic plotline: four key stages that are organized around the concept of the “lockdown,” the primary strategy adopted by the government to manage the spread of the virus (see Figure 1). The acts to this lockdown drama are described below:

### Evading Lockdown

For Rosenberg (p. 4), the “progressive revelation” of an epidemic ensures that denial characterizes the first stage of an epidemic: “bodies must accumulate…before officials acknowledge what can no longer be ignored.” The UK government's response followed a similar pattern: through late-February and early-March, it came under increasing pressure to act as cases in nearby countries expanded exponentially. The response, released on March 3rd (2), was to evade draconian measures and instead “contain, delay, research, and mitigate.” Evasion was based on an understanding of individual rather than collective behavior during emergencies (3). Firstly, the idea of “behavioral fatigue” was used to argue that a lockdown would not be effective because it would be unacceptable to the public, who would become tired of restrictions and behave in potentially hazardous ways (4). Secondly, the idea of “herd immunity” was used in cautioning against a full lock-down. The Prime Minister announced that a balanced approach to protecting the National Health Service (NHS) would mean some people would have to take coronavirus “on the chin.” More scientifically, the government's Chief Scientist suggested that herd immunity would broaden and flatten the epidemic peak. Individual responsibility and a sense of duty to “do the right thing” was tasked with defeating Covid-19. Thus, rather than government imposed containment measures, such as banning mass gatherings and closing schools, it was members of the public who took these decisions.

### Entering Lockdown

If turning to “rational understanding of phenomenon in terms that promise control,” represents the next stage in Rosenberg's plotline (p. 5), this was made palpable in the UK's adoption of lockdown measures by the release of epidemiological modeling in mid-March (5). These models estimated that the containment approach would lead to 250,000 deaths (6). A week later, the lockdown was announced, with policymakers emphasizing that lockdown decisions were reliant on “the science” and the rate of infection (known as the R number). The message to the public was clear: “stay home, protect the NHS, save lives.” The approach reflected a dramatic shift away from relying on individual freedom, and highlighted the government's centralized scientific infrastructure involved in controlling disease. Whilst the Scientific Advisory Group for Emergencies (SAGE) and its sub-groups like the Scientific Pandemic Influenza Group on Modeling (SPI-M) had been advising the government since the start, these scientists appeared at daily press briefings, and their advice deferred to in the exclusionary narrative of “the science”. Devolved approaches fared less well, reflected in the abandoning of localized test and trace methodologies that had worked well in other countries (6).

### Enduring Lockdown

Accompanying this rational understanding, the third act of an epidemic involves routines and rituals and the imposition of “familiar frames of explanation and logically consequent policies” (Rosenberg, p. 7). Throughout the UK's lockdown, a daily government briefing became a scientific stage for “the science” and the “R number” to reassure the public of the government's strategy [cf. (7)]. Targets were set to recruit 18,000 contact tracers, to test 100,000 people a day and to supply millions of pieces of personal protective equipment (PPE). Back-stage the reality was messier with double-counting of tests creating what leading statistician Professor Sir David Spiegelhalter called “pure number theater.” If this dented public confidence in the government's handling of the pandemic, it was a mere foretaste. Firstly, a change in messaging from “stay home” to “stay alert” created confusion amongst the public. Secondly, the UK's former chief scientific advisor, David King, established an “Independent SAGE,” with a more diverse scientific membership, to address criticisms of the lack of scientific transparency and trustworthiness. Then, news broke that Dominic Cummings - the Prime Minister's chief advisor - and his family had broken rules. Public trust in government plummeted, the devolved governments in Scotland and Wales emphasized their differences, and Cummings was used by the public to justify breaking lockdown rules.

### Exiting Lockdown

Whilst epidemics may end with a whimper, their ending also prompts moral judgment: to ask if the “dead have died in vain?” (Rosenberg, p. 9). The ending of the lockdown, began on May 13th, reaching its zenith on “super Saturday” when English pubs reopened on July 4th. Yet this stage is also marked by ambiguity, for example through increasing organizational complexity. This includes the establishment of a Joint Biosecurity Centre, to advise on the UK's coronavirus “alert levels” as part of a new Covid-19 alert system. Chaired by a member of the security services, Covid-19 is reframed as a matter of security and its relationship to existing public health infrastructure is unclear. Organizational complexity is demonstrated too by the reliance on a range of private organizations (such as Serco) to deliver contact tracing or create contact tracing apps. As scientists took a backseat following their daily appearances, politicians took control of the recovery, seeking to “build back better” and restore the economy. The specter of a second-wave, super-spreading events in abattoirs and local lockdowns, suggests the final curtain is yet to fall.

## The Anticipatory Logic of Animal Health

If epidemics like Covid-19 follow familiar plotlines, can it be described as unexpected and unprecedented? If the Covid-19 epidemic narrative reflects institutional forms and cultural assumptions, it also reflects how understandings of disease control are too narrowly framed and ignore important lessons from the management of animal disease in the UK over the last 20 years. The outbreak of Foot and Mouth Disease (FMD) in the UK in 2001, for example, focused government attention on preparedness planning, not least because the inability of the government to handle such an outbreak had already been predicted (8). As Anderson (9) argues, “precaution, preemption, and preparedness” have become obsessions, giving rise to “anticipatory logics,” and practices of calculating the future to instill resilience across government organizations and responsible conduct amongst the public. Bearing witness to the management of animal disease - its social practices and consequences - can be seen as an anticipatory logic itself. Indeed, as the discourse of “One Health” suggests (10), there should be much to learn and apply from animal to human disease management. For the narrative arc of Covid-19, what would this anticipatory logic have told us, and potentially pre-empted?

Firstly, arguments over the role of epidemiological modeling should be expected because of the way space, subjectivity and politics are encoded within it. The experience of FMD in 2001 highlighted different political choices on which to base decisions. For some, a pre-emptive cull of animals was not only illegal, but socially and economically regressive due to the abstract nature of modeling (11). Other studies of FMD modeling have pointed to the geographical disconnect between computer modelers in distant cities, compared with the situated and nuanced understandings of other experts (such as field veterinarians) whose connection with place provides a different understanding of disease transmission (12). These differences are also tied to spatial styles for governing: command and control is associated with governing from a distance using models that treat space as universal and knowledge as mobile (13). By contrast, devolved approaches are associated with proximate experts and expertise that is situated and variable. Clearly, these distinctions are disciplinary as well as spatial. Thus, different epidemiological subjectivities are endorsed and/or marginalized by choices made by governments when managing disease (14). The management of Covid-19 displays the same pattern: command and control through modeling and the marginalization of local and regional health knowledges. In animal health, the effect of this disciplinary and social marginalization can have long-lasting effects. These studies also point to a better future that recognizes how epidemiological knowledge is not bounded but created in a borderland in which approaches overlap (15) and by integrating participatory forms of modeling (16), more inclusive forms of disease control can be developed.

Secondly, the collapse of trust in the UK government's approach to governing Covid-19 was foretold through the management of animal disease. Starting with Bovine Spongiform Encephalopathy (BSE), government failures in communicating scientific uncertainty (17) have contributed to a lack of public confidence in the handling of disease. BSE was not an isolated incident: the public were similarly alarmed by the handling of FMD (18), whilst farmers were similarly distrustful of attempts to manage bovine Tuberculosis (bTB). Distrust may stem from the contrast between different forms of understanding disease and the distinctions between scientific and experiential knowledges (19). As Cassidy (20) describes, recourse to “big science” as a means of resolving disputes that rest on values rarely succeeds and often has the opposite effect. Part of the problem here is communicating the distinction between population and individual medicine and the creation of what Rose (21) calls “the prevention paradox.” As studies of animal disease show, where population disease interventions fail to correspond to individual experiences, exceptions to the rules, and conflict with cultural norms drives mistrust of government and fatalism. For Covid-19, the reliance on the R number has the same problems. Not only does it misrepresent that epidemics are multiple and vary between sites (e.g. community, hospital and care homes), but the universal presentation fails to reflect how the public have a geographically nuanced understanding of disease risks and transmission (22).

Thirdly, the challenges of creating testing regimes and technologies to track and trace infections are well-understood within studies of animal disease and agriculture. The extent to which testing can deliver on promises set for it will reflect its social organization. For example, in the management of bTB, who conducts tests has come to reflect broad political-economic choices that have infiltrated the management of animal disease. Presumed efficiencies of the private sector have led to the contracting out of disease surveillance but this has not been without consequences. The close “relational distance” between farmers and their own veterinarians paid by government to regulate their clients has raised questions over the “accuracy” of interpretation of test results, as a result of testers acting as field-level epidemiologists and taking local factors into account (23). Similarly, for Covid-19, if test results are to trigger the use and commitment to new track and trace technologies, then these will rely on more than just test results. As Higgins et al. (24) show, acting on biosecurity information involves a different set of behavioral logics than those that are imagined by regulators. The cultural expectation of what counts as “good farming” and the “good farmer” can undermine official guidance on avoiding animal disease or disclosing suspicious symptoms (25, 26). Shaping conduct by governing through individualistic biosecurity subjectivities (27) written into official documents and technologies has limits: use of biosecurity practices and reporting of suspicious deaths and sightings is not simply a matter of “staying alert,” but is emergent from a complex relationship of social, economic and environmental relationships (28–31).

Finally, studies of the management of animal disease highlights the mobility of disease experts and expertise. Whilst the psycho-social impacts of eradicating animal disease upon animal disease experts (32, 33) may foretell how medical doctors and health care staff will respond to their own trauma of treating Covid-19, one likely response will be to exit the profession or migrate to other countries as a form of recovery (34). In fact, whilst the UK's initial approach to managing Covid-19 through herd immunity may reflect a form of “British Exceptionalism,” animal disease management has recently been anything but international. Policy documents clearly reflect the international spread of logics and technologies of disease management, such as the neoliberal forms of responsibilization and cost-sharing and its technologies of risk-based trading developed in Australia and New Zealand. Nevertheless, whilst the global flow of ideas, experts and expertise appears to continue to shape how disease control is imagined, it is equally true that the globalization of disease regulations has not been met without resistance, as politicians seek to protect their own interests (35, 36). In this sense, in the face of global consensus over the appropriate tools and methods to deploy, the UK's approach finds some precedent in the management of animal disease.

## Conclusion: Whose Failure?

In traversing Covid-19's narrative arc, we wish to make three related points. The first is that it seems that social studies of animal disease provide a mirror of clarity to the narrative arc of Covid-19. If paying attention to the management of animal disease provides an “anticipatory logic,” it seems to be one worth paying attention to in order to provide the kind of “situational awareness” required to help prevent mistakes from being made in future pandemics. Social studies of animal disease add to the “ecology of knowledges” that are required to resolve problems where “the facts are uncertain, the social stakes are high, decisions are urgent and values are in dispute” - what Funtowicz and Ravetz [(37), p. 744] define as “post-normal science.” The warnings and advice that social studies of animal disease can signal may therefore help to broaden institutions “sense-making” capabilities, providing different perspectives and alternatives, and as Weick (38) puts it, to drop familiar tools and develop new ones.

Secondly, there is also a broader lesson for the kinds of social science that can be used here too. One difference between the handling of FMD in 2001 and Covid-19 has been the rise of behavioral science. The pandemic has provided an opportunity for behavioral scientists to reframe disease management as a behavioral problem and claim a place alongside epidemiologists. Their claims of expertise have, however, routinely ignored the social science of animal disease. Thus, Bavel et al. (39) review of the role of social science in managing Covid-19 ignores social research on the human dimensions of managing animal disease. Equally, there is a danger that the social sciences have been narrowly framed: aligned with disciplining the individual perspective of “nudge” behavioral economics rather than acknowledging community action (3). Alternatively, these attempts to provide social scientific certainty, ignore the messy realities of disease and the need to understand the kinds of social work required to make disease control possible (40).

This narrow definition leads to our final question: why have lessons from animal disease studies been ignored? This seems all the more apposite given the extent to which the discourse of “One Health” has become ubiquitous in anticipation of the next pandemic (41). In response to Covid-19, was it most appropriate for veterinary experts to help on the front line of the human medical crisis, donate their PPE from the sidelines, or in the face of a labor crisis, to focus on those dimensions of health (such as veterinary public health) that their specialism allowed? With Chief Veterinary Officers suggesting the latter, the experience of Covid-19 seems to speak to the broader limitations of the One Health movement, or at least, reinforce a demarcation and segregation between its various components. Indeed, social scientific studies of One Health already reveal the extent to which understandings of even an epidemic are socially constructed, distributed and laden with power relations (42, 43). Or, as Hinchliffe [(40), p. 28] suggests, visions of One Health can reduce complexity by focussing narrowly on contamination and transmission, thereby effacing the “local, contingent and practical engagements that make health possible.” Rather than this version of One Health, argues Hinchliffe, what is preferable is a version that understands the social work that is required to make health work within increasingly complex disease ecologies. Whilst social studies of animal disease offer an immediate mirror into new and emerging infections like Covid-19, it is toward this longer lasting social understanding of health that might be its greatest contribution.

## Data Availability Statement

The original contributions presented in the study are included in the article/supplementary material, further inquiries can be directed to the corresponding author/s.

## Author Contributions

GE prepared the main draft. DM contributed material and ideas and edited the text.

## Conflict of Interest

The authors declare that the research was conducted in the absence of any commercial or financial relationships that could be construed as a potential conflict of interest.
